# A Case of Violent Uterine Hemorrhage, Occurring Twelve Days after Delivery

**Published:** 1855-12

**Authors:** N. B. Drewry

**Affiliations:** Erin, Ga.


ARTICLE II.—*A case of Violent Uterine Hemorrhage, occurring twelve days after Delivery.* By N. B. DREWRY, M. D., of Erin, Ga.

On the morning of the 11th of September last, I was called to see Mrs. ——; age 23 years; stout; of full and plethoric habit, and of amiable disposition; whom I found in labor with her second child. The labor had been going on some time when I arrived, and continued to progress, with a favorable presentation, (1st vertex of Meigs and Dewees,) till she was safely delivered of a living and healthy child. The delivery was completed in about four hours after my arrival; and in due time after the birth of the child the placenta was removed. Not more than the usual amount of hemorrhage occurred, nor indeed so much as is often found after delivery. I left her, quiet and comfortable, early in the afternoon of the same day, and to all appearance doing well. Nothing unusual in her case came to my knowledge, until on the night of the 22nd, eleven days after her confinement, when I was summoned, in great haste, to see her, whom the messenger reported "very sick." On my arrival, at 1 A. M., of the 23rd, I found her literally bathed in blood, and profuse hemorrhage still going on. The statement I received from the lady and her husband was, that no untoward symptom had occurred since her delivery on the 11th, until a few hours before my arrival; that at the usual time of retiring she felt as well as at any evening previously, and that she felt no symptom of such an accident, until awoke by the excessive discharge of blood from the vagina. Being greatly alarmed at the condition in which she found herself on awaking, she immediately attempted to rise up in bed. The husband being aroused, was also alarmed at her situation and despatched a message for me. After getting this history of the case, I proceeded to an examination of the symptoms, and found her very faint, and the discharge from the uterus continuing. From the emergency of the case, I had her hips raised on a level with, or higher than her head, a position most likely to favor the suppression of the hemorrhage by the force of gravity. This was intended to aid in filling two very important indications in the case; firstly, to prevent further loss of blood; secondly, to restore the deficiency that existed in the head, so as to prevent syncope. From the length of time that elapsed after delivery before the hemorrhage occurred, and from the absence of any unfavorable symptom in the interim, I was no little puzzled to divine the cause of so sudden and unexpected an attack. By a vaginal examination, it was ascertained that the os and cervix uteri were relaxed in some degree, and soft; that the discharge was of a scarlet hue, such as would likely proceed from an artery.

The treatment, after placing the patient in the most favorable position, consisted, at first, of cold water applied externally to the pelvic region; cold water injections in the vagina, and internally Plumbi Acetus.

Under this treatment the improvement seemed not sufficient to warrant its continuance. The ergot of rye was then brought into requisition, and the Vinum Ergotœ in the dose of six fluid drachms was given. This quantity was increased and repeated, at suitable intervals, until three fluid ounces had been taken. With the use of this medicine, the hemorrhage ceased entirely about 5 A. M., four hours after I arrived.

My patient, though feeble and exhausted, slowly but gradually improved by careful management, and had no recurrence of the hemorrhage.*

---

*This case, though occurring at a much later period after delivery than usual, was doubtless inertia of the uterus, which generally occurs shortly after the birth of the child, giving rise to violent hemorrhage; and, as in this case, it will generally be found, that those means which best promote the contraction of the womb will be most effectual.—EDS.

